# Sixteenth Anniversary Meeting of the Illinois State Medical Society

**Published:** 1866-07

**Authors:** S. A. McWilliams

**Affiliations:** Sec’y pro tem.


### SIXTEENTH ANNIVERSARY MEETING OF THE ILLINOIS STATE MEDICAL SOCIETY.

DECATUR, JUNE 5, AND 6, 1866.

The regular Annual Meeting of the Illinois State Medical Society assembled in Powers' Hall, Decatur, June 5th, at 9½ o'clock A.M. The President, Dr. J. M. STEELE, of Grandview, called the Society to order, and the Rev. D. C. MARQUIS, opened the exercises by prayer.

Dr. E. W. MOORE, Chairman of the Committee of Arrangements, welcomed the Delegates to the hospitalities of the City of Decatur; and reported as present the following members:—

Drs. J. H. Hollister, J. S. Jewell, E. L. Holmes, J. W. Freer, N. F. Quales, Ira Hatch, T. D. Fitch, and S. A. McWilliams, of Chicago, Cook Co.

Drs. T. F. Worrell, D. L. Crist, C. R. Parke, John Little, S. W. Noble, and D. O. Crist, of Bloomington, McLean Co.

Drs. J. A. Heckelman, Alex. McBride, S. McBride, S. T. Trowbridge, B. A. Allison, J. A. W. Hostetter, O. F. Parker, Ira N. Barnes, E. W. Moore, and J. Brown, of Decatur, Macon Co.

Drs. Daniel Stahl, Louis Watson, Joseph Robbins, W. M. Landon, of Quincy, Adams Co.

Drs. Geo. T. Allen, P. J. Wardner, and A. A. Patterson, of Springfield, and W. H. Veatch, of Pawnee, Sangamon Co.

Drs. Geo. R. Bibb, David Prince, of Jacksonville, and R. E. McVey, of Waverly, Morgan Co.

Dr. E. W. Mills, of Sullivan, Moultre Co.

Drs. J. H. Tyler, B. K. Shurtleff, R. T. Richards, and W. W. Adams, of Clinton, DeWitt Co.

Dr. H. Noble, of Heyworth, McLean Co.
" N. Wright, of Chatham, Sangamon Co.
" Geo. W. Albin, of Neoga, Cumberland Co.
" F. B. Haller, of Vandalia, Fayette Co.
" J. M. Steele, of Grandview, Edgar Co.
" L. T. Hewins, of Loda, Iroquois Co.
" S. P. Breed, of Princeton, Bureau Co.
" W. M. Chambers, of Charleston, Coles Co.
" John Wright, of Clinton Institute of Medicine.
" J. O. Hamilton, of Jerseyville, Jersey Co.

The following were then proposed and duly elected permanent members:—

Drs. J. M. Harwood; W. E. Wilson; B. A. Allison; Charles N. Denison; W. B. Hostetter; O. F. Parker; J. A. Heckelman; and W. H. Walters, of Decatur, Macon Co.

Drs. P. H. Bailhache; H. C. Barrell; B. M. Griffith; and H. H. Roman, of Springfield, Sangamon Co.

Dr. J. N. Ralston, of Quincy, Adams Co.
" C. Gorham, of York,
" W. M. Wilcox, of Matoon,
" S. B. McGlumphy, of Lincoln,
" D. T. Kyner, of Macon,
" J. P. Taggert, of Cairo,
" C. J. Gill, of Bloomington,
" J. A. Ward, of Livingston,
" N. G. Blalock, of Mount Zion, Macon Co.

A letter from Dr. N. S. Davis, Permanent-Secretary, was read, stating that he was unable to attend the present Annual Meeting, on account of severe sickness.

On motion, Dr. S. A. McWilliams, of Chicago, was appointed Secretary *pro tem.*

The following committee of one from each County represented, was appointed to nominate the Officers, and fill the Standing Committees, for the ensuing year, viz.:—

Dr. H. Noble, of McLean Co.
" Louis Watson, of Adams Co.
" S. P. Breed, of Bureau Co.
" J. W. Freer, of Cook Co.
" W. M. Chambers, of Coles Co.
" G. W. Albin, of Cumberland Co.
" J. H. Tyler, of DeWitt Co.
" J. M. Steele, of Edgar Co.
" F. B. Haller, of Fayette Co.
" L. T. Hewins, of Iroquois Co.
" S. T. Trowbridge, of Macon Co.
" R. E. McVey, of Morgan Co.
" N. Wright, of Sangamon Co.
" S. B. McGlumphy, of Logan Co.

The Committee retired, and after a brief consultation reported the following nominations:—

*For President*—Dr. F. B. Haller, of Vandalia,
*Vice-President*—Dr. L. T. Hewins, of Loda,
*2d Vice-President*—Dr. N. Wright, of Chatham,
*Treasurer*—Dr. J. H. Hollister, of Chicago.

On motion, the report of the Committee was accepted, and the candidates nominated unanimously elected.

Drs. W. M. Chambers and H. Noble were appointed a committee to conduct the officers just elected to their places.

Dr. J. M. Steele, the retiring President, thanked the Society for its uniform kindness and support, and retired with the cordial good wishes of every member.

Dr. F. B. Haller, the President elect, was then introduced, and, after a brief and appropriate address, entered upon the discharge of his official duties.

A communication from the Adams County Medical Society was presented and read, making certain charges against Dr. Addison Niles, of Quincy.

After some discussion, the following resolution, offered by Dr. T. F. Worrell, of Bloomington, was adopted, viz.:—

*Resolved,* That a committee be appointed, who shall prefer charges against Dr. Addison Niles, based on the representations made by the Adams County Medical Society, and shall furnish him with a copy of such charges, with notice to appear before this Society, at it next regular Annual Meeting.

Drs. T. F. Worrell, of Bloomington, J. Brown, of Decatur, and H. Noble, of Heyworth, were appointed such committee.

The following Report of the PermanentSecretary and Publishing Committee was then read:—

REPORT OF THE PERMANENT SECRETARY AND COMMITTEE OF PUBLICATION, FOR 1866.

In accordance with the Constitution, which provides that the State Medical Society shall hold its Annual Meeting on the first Tuesday in June, whenever the American Medical Association meets on the first Tuesday in May, the Permanent-Secretary gave early notice, in both the Medical Journals published in the State, that the present Meeting would be held at Decatur, on the first Tuesday in June. Also, that, in consequence, the Delegates appointed to attend the American Medical Association in Boston, last year, must also fill the same appointment at Baltimore, this year, as there would be no opportunity to appoint their successors. He furnished each Delegate new credentials for that purpose.

In accordance with the directions of the State Society, at its last Annual Meeting, the Committee of Publication put the Transactions of the last year to press early, and as usual printed 300 copies; also, separately, 500 copies of the Constitution, By-Laws, and Code of Ethics.

Although the whole expense of type-setting for the Transactions was avoided by first publishing the matter in the *Examiner*, yet the printer's bill, for the present year, is as follows:—

| | |
|---|---|
| 300 Copies of Transactions for 1865, --------- | $195,49 |
| 500 do of Constitution, By-Laws, etc., ---- | 57,00 |
| Total, --------------------- | $252,49 |

The amount paid on this bill and the balance unpaid, will be shown by the Report of the Treasurer.

Of the 300 copies of Transactions, about 40 were required to supply Members of the Society, about 20 have been sent to the various Medical Periodicals, leaving about 240 copies on hand. About the same proportion of those published each year, since the undersigned has been Secretary, are still left to encumber the shelves of his office. In consequence of the very small number of copies of Transactions either paid for or read by the Members of the State Society, and the constant indebtedness of the Treasury, we would respectfully suggest that, in future, the Committee of Publication be instructed to publish the Proceedings of the Society in the *Chicago Medical Examiner*, and issue extra copies to the number only just sufficient to supply such Members of the Society as have paid their dues before the 1st of August. The *Chicago Medical Journal* might also publish so much of the Proceedings as its Editors might choose. By this course, the Society would soon be out of debt, and receive all the real benefits that have ever been obtained by the publication of their Proceedings.

All of which is submitted. N. S. DAVIS,
*Permanent-Secretary.*

After having been read, the Report was referred to a Special Committee, consisting of Drs. S. W. Noble, W. M. Chambers, and T. F. Worrell.

The report of Dr. J. H. Hollister, as Treasurer, was read and referred to an auditing committee.

The Treasurer also reported, that in accordance with the instructions of the Society, he had caused to be prepared a book, in which to preserve the constitution, by-laws, list of officers and members, and the Treasurer's accounts.

The Society then adjourned until 2 o'clock P.M.

### AFTERNOON SESSION.

The Society was called to order at 2 o'clock P.M., Dr. F. B. Haller, President, in the Chair.

Dr. H. Noble, Chairman of the Committee on Practical Medicine, presented his report, with an accompanying paper from Dr. F. B. Haller. After reading, the report and accompanying paper were referred to the Committee of Publication.

Dr. E. L. Holmes, Committee on Ophthalmology, presented and read his annual report, which was accepted and referred to the Committee of Publication.

On motion, it was resolved that the annual assessment on each member for the present year be *three dollars.*

The Committee to whom had been referred the Annual Report of the Permanent-Secretary, made the following report:—

Your Committee, to whom was referred the report of Dr. N. S. Davis, would respectfully report that, in our opinion, the publication of the proceedings of this Society in either of the medical journals would be inexpedient.

We, therefore recommend, that the proceedings be published in pamphlet form, and that there be but one hundred copies published.

We further recommend that the Committee of Publication have the privilege of first publishing the proceedings in the Medical Examiner, to save expense—provided there is not money enough in the treasury to pay for the immediate publication.

We likewise recommend, that the annual assessment be three dollars, and that the Treasurer correspond with every delinquent member, explaining his manner of keeping the records, and urging them in the strongest manner possible to keep themselves good on the books.

All of which is very respectfully submitted.

S. W. NOBLE,
W. M. CHAMBERS,
T. F. WORRELL.

After considerable discussion, on motion of Dr. D. Prince, the report of the Committee was laid on the table.

Dr. D. Prince proposed, as an amendment to the clause of the constitution relating to permanent members, that the words "but without the right of voting," be stricken out. Proposition lies over one year, under the rule.

On motion, the Society adjourned until 8 o'clock P.M.

EVENING SESSION.

At 8 o'clock P.M., the Hall was well filled with a popular audience, to hear an address from Dr. GEO. T. ALLEN, of Springfield. The President called the meeting to order, and the speaker was introduced by Dr. E. W. MOORE, Chairman of the Committee of Arrangements.

The address of Dr. Allen was listened to with much pleasure and profit. At its close, the Society adjourned until 9 A.M. of Wednesday.

WEDNESDAY, SECOND DAY.

The Society met at 9 o'clock A.M., the President, Dr. HALLER, in the Chair.

The minutes of the previous day's proceedings were read by the Secretary and approved.

On motion of Dr. J. BROWN, the Society tendered a vote of thanks to Dr. Allen for his public address last evening.

Dr. J. H. HOLLISTER presented and read a paper on Bromine and its compounds, as remedial agents, and the pathological conditions indicating their use. The paper elicited some discussion, and was referred to the Committee of Publication.

Dr. J. S. JEWELL presented and read a full and carefully prepared report on Cerebro-Spinal Meningitis, which was listened to with close attention, and referred to the Committee of Publication.

On motion, the Society adjourned to 1½ o'clock P.M.

AFTERNOON SESSION.

The meeting was called to order at 1½ o'clock P.M., the President, Dr. HALLER, in the Chair.

The Secretary read a paper on Spotted Fever, by Dr. F. R. PAYNE, of Marshall, which was referred to the Committee of Publication.

Drs. Worrell and Brown, from the committee appointed to prefer charges against Dr. Addison Niles, of Quincy, reported as follows:—

"The undersigned having been appointed a committee to prefer charges against Dr. Addison Niles, of Quincy, a member of this Society, respectfully submit the following report:—

"That we charge Dr. Niles with having violated the Code of

Ethics, by representing the 'Quincy Medical Society,' which we consider irregular, being composed, in part, of members who had been expelled from the Adams County Medical Society.

T. F. WORRELL,
J. BROWN."

The report was accepted, and the Secretary instructed to furnish Dr. Niles with a copy, and request him to answer to the same in person at the next annual meeting of the State Medical Society.

Dr. H. NOBLE, Chairman of the Committee on Nominations, made the following report:—

*For Place of Next Annual Meeting.*—SRINGFIELD.

*For Assistant-Secretary.*—Dr. P. H. Bailhache, of Springfield.

*Committee of Arrangements.*—Drs. N. Wright, of Chatham; B. M. Griffith, H. B. Buck, J. Townsend, and Wm. Jayne, of Springfield.

*Committee on Practical Medicine.*—Drs. J. Adams Allen, of Chicago; R. E. McVey, of Waverly; and L. T. Hewins, of Loda.

*Committee on Surgery.*—Drs. H. W. Davis, of Paris; C. R. Parke, of Bloomington; and G. R. Bibb, of Jacksonville.

*Committee on Obstetrics.*—Drs. DeLaskie Miller, of Chicago; N. Wright, of Chatham; and J. N. Ralston, of Quincy.

*Committee on Drugs and Medicines.*—Drs. P. H. Bailhache, of Springfield; N. T. Quales, of Chicago; and J. Miner, of Waverly.

*Committee on Ophthalmology.*—Dr. E. L. Holmes, of Chicago.

*Committee on Curvatures of Spine and Hip Disease.*—Drs. J. W. Freer and R. G. Bogue, of Chicago.

*Committee on Plastic Surgery.*—Dr. David Prince, of Jacksonville.

*Committee on the Co-Relation between Electricity and Nervous Force.*—Dr. D. T. Kyner, of Macon.

On motion, the report of the Nominating Committee was accepted, and its recommendations adopted.

On motion of Dr. T. D. FITCH, a committee of three was appointed, to report at the next annual meeting, on "Specialties and Medical Advertising."

The President appointed Drs. T. D. Fitch, N. S. Davis, and D. Prince, to act as such committee.

A paper on the use of Bromide of Ammonium in the Treatment of Epilepsy, by Dr. IRA HATCH, of Chicago, was read by Dr. Hollister, and referred to the Committee of Publication.

Dr. D. PRINCE proposed the following amendment to the constitution:—

"*Art. 3d—Meetings.*

"The Annual Meetings of this Society shall be held on the *Third* Tuesday in May of each year."

On motion, the following members were duly elected Delegates to the next Annual Meeting of the American Medical Association:—

Dr. F. B. HALLER, of Vandalia,
" DAVID PRINCE, of Jacksonville,
" J. H. HOLLISTER, of Chicago,
" LOUIS WATSON, of Quincy,
" H. NOBLE, of Heyworth,
" T. F. WORRELL, of Bloomington,
" S. T. TROWBRIDGE, of Decatur,
" JOHN WRIGHT, of Clinton,
" P. J. WARDNER, of Springfield,
" D. L. CRIST, of Bloomington,
" J. M. STEELE, of Grandview,
" J. A. W. HOSTETTER, of Decatur,
" L. T. HEWINS, of Loda,
" G. W. ALBIN, of Neoga,
" WM. M. CHAMBERS, of Charleston,
" C. GOODBRAKE, of Clinton,
" S. W. NOBLE, of Bloomington,
" SAMUEL MCBRIDE, of Decatur,
" JAMES MINER, of Waverly,
" J. BROWN, of Decatur,
" GEO. T. ALLEN, of Springfield,
" R. T. RICHARDS, of Santa Anna,

Dr. J. Robbins, of Quincy,
" R. E. McVey, of Waverly,
" W. E. Wilson, of Decatur.

On motion of Dr. Hollister, each delegate was authorized to appoint a substitute, in case of his own inability to attend.

Dr. Clarke, of Illiopolis, exhibited an interesting specimen of monstrosity.

Dr. S. P. Breed, of Princeton, exhibited a uterine polypus, with a verbal history of the case; for which he received the thanks of the Society.

Dr. E. W. Moore, Chairman of the Committee of Arrangements, presented a list of volunteer papers.

Dr. C. R. Parke, of Bloomington, read the report of a case of death from the inhalation of chloroform; which, after an interesting discussion, was referred to the Committee of Publication.

Dr. S. T. Trowbridge, of Decatur, presented a communication in the form of a law to be enacted by the State Legislature, designed to secure a higher grade of qualifications for practising physicians in this State.

On motion of Dr. Stahl, of Quincy, the paper of Dr. Trowbridge was referred to the following Committee:—

Drs. S. T. Trowbridge, Geo. T. Allen, P. H. Bailhache, H. Noble, and David Prince; and the same instructed to prepare suitable measures of legislation, and to urge their passage by the next Legislature.

Dr. N. Wright, of Chatham, read a paper on Cholera, which was referred to the Committee of Publication.

Dr. Veatch presented a paper on Malarial Fever, which was referred to the Committee of Publication.

Dr. Geo. T. Allen, of Springfield, was appointed a Special Committee to report on the *Radical Cure of Reducible Hernia.*

Dr. W. M. Chambers, of Charleston, was appointed a Special Committee to report on Syphilis.

On motion of Dr. S. W. Noble, it was resolved that, at the next Annual Meeting, all volunteer papers be handed to the Chairman of the Committee of Arrangements, at the commencement of the meeting.

On motion of Dr. HOLLISTER, a vote of thanks was unanimously tendered to the Committee of Arrangements, and to the Physicians of Decatur, for the very judicious and hospitable manner in which they had received and entertained the Society at its present meeting.

On motion, the Society adjourned *sine die.*

The number in attendance at the present meeting was larger than at any previous meeting since the organization of the Society. S. A. McWILLIAMS, *Sec'y pro tem.*

